# Trends in antimicrobial management of gonorrhoea by general practitioners in Amsterdam, the Netherlands, between 2010 and 2016: a cross-sectional study

**DOI:** 10.1186/s12875-018-0900-9

**Published:** 2019-01-15

**Authors:** Roos van Amerongen, Roel P. Gazendam, Jan E. A. M. van Bergen

**Affiliations:** 1 Department of General Practice, Amsterdam University Medical Center, University of Amsterdam, Meibergdreef 9, P.O. Box 22700, 1100 DE Amsterdam, The Netherlands; 2 STI AIDS Netherlands (Soa Aids Nederland), Amsterdam, the Netherlands; 3 National Institute for Public Health and Environment (RIVM), Epidemiology & Surveillance Unit, Centre for Infectious Disease Control, Bilthoven, the Netherlands

**Keywords:** Gonorrhoea, Resistance, Antimicrobial management, General practice, Ceftriaxone

## Abstract

**Background:**

Sexually transmitted infections (STI) caused by multidrug resistant *Neisseria gonorrhoea* are an emerging threat to global health. In the Netherlands, the general practitioner (GP) provides the major part of STI care. In 2013 an update of the Dutch guideline was published, recommending a single dose of intramuscular ceftriaxone as treatment for gonorrhoea infections. Data from a Dutch General Practitioner research database was used to investigate the guideline implementation for the treatment of gonorrhoea. A survey was conducted to gain more insight in GPs experiences with the recommended intramuscular therapy.

**Methods:**

Data on STI-related episodes and STI-diagnoses for gonorrhoea, based on ICPC codes were obtained from the electronic medical records (EMRs) from 35 GPs in Amsterdam for the years 2010 to 2016. Questionnaires regarding the treatment preferences were sent to GPs participating in the research network database.

**Results:**

The number of gonorrhoea cases treated with first choice therapy increased from 81% in 2010 (intramuscular cefotaxime or ceftriaxone) to 93% in 2015 (only cefttriaxone). The number of ceftriaxone prescriptions increased substantially from 30% in 2010 to 93% in 2015. GPs preferred a single intramuscular shot of a third-generation cephalosporin above multiple oral doses of other antibiotics.

**Conclusions:**

The results demonstrate a successful shift in the antimicrobial management of gonorrhoea infections to ceftriaxone monotherapy according to the national guideline. GPs in this higher prevalence area in Amsterdam reported limited barriers in the intramuscular administration of third-generation cephalosporins.

**Electronic supplementary material:**

The online version of this article (10.1186/s12875-018-0900-9) contains supplementary material, which is available to authorized users.

## Background

The incidence of sexually transmitted infections (STI) caused by *N. gonorrhoeae* has increased worldwide in the last decade [1]. The vast development of multi-resistant gonococcal strains and treatment failures are an emerging threat to global health [1, 2]. International guidelines and most European countries recommend dual antimicrobial therapy (ceftriaxone and azithromycin) as empirical blind treatment option in the first line [3–5]. In the Netherlands, dual therapy with azithromycin is only advised if a Chlamydia trachomatis coinfection is suspected or diagnosed [6]. Since it is uncertain whether dual therapy has additional benefit in the control of antimicrobial resistance compared with monotherapy, the Dutch STI guideline recommends monotherapy with an intramuscular dose of a third-generation cephalosporin [7]. In 2004, the Dutch STI guideline recommended intramuscular cefotaxime as gonorrhoea treatment in general practice. From 2013 onwards, intramuscular ceftriaxone was described as first choice therapy because of a more favorable pharmacokinetic profile [6]. So far, few cases of ceftriaxone-resistant gonococcal strains have been identified [8, 9].

The threat of untreatable gonorrhea indicates the need for correct antimicrobial management. In the Netherlands, the major part of STI care is provided by GPs [10]. A previous report using a Dutch national database, containing data of almost 500 general practices, studied GPs’ prescriptions in the Netherlands between 2005 and 2010 [11]. The study demonstrated that 5 years after an update in the Dutch STI-guideline –including a change in preferred medication for gonorrhoea to an intramuscular third-generation cephalosporin- still only 64% of the patients with a gonorrhoea infection were treated according to the new guidance and ciprofloxacin was still commonly used.

So far, implementation of the current guideline, published end of 2013, has not yet been investigated. Neither are the factors that may lead to the administration of other (oral) drugs instead of the recommended first choice intramuscular antibiotics. Alternative treatments could be based on culture outcomes with susceptibility patterns, although the general shift in diagnostic methods from culture-based to nucleic acid amplification test (NAAT), has limited this approach [12]. Other factors may also contribute to alternative administration, such as GPs experiences with the administration of intramuscular cephalosporins.

We investigated the implementation of the updated Dutch STI guideline and explored experiences with the recommended intramuscular therapy among GPs in Amsterdam, the Netherlands, between 2010 and 2016.

## Methods

### Design

A cross-sectional study was conducted to collect data on gonorrhoea cases (including all anatomic locations) and antimicrobial management in general practice. The experiences of GPs with the recommended intramuscular administration of third-generation cephalosporins were investigated by a questionnaire.

### Setting

The study was performed using the General Practice Department research database of the Amsterdam University Medical Center (AUMC) in southeast-Amsterdam, the Netherlands. Southeast-Amsterdam is a multicultural and low economic status neighborhood with a relatively high prevalence of STI [13]. The database contains anonymized electronic health records of more than 45,500 active patients from 35 GPs in six health centers in southeast-Amsterdam. These data include sex, date of birth, information on patients’ medical history, summaries of consultations, results of diagnostic tests and drug prescriptions. The health problems of the patients are documented as episodes and coded according to the International Classification of Primary Care-1 (ICPC-1).

### Data collection

A 6-year period from January 1st 2010 to January 1st 2016 was assessed to determine potential gonorrhoea cases. Details were obtained about all registered episodes coded as gonorrhoea according to the ICPC-1, i.e. Y71 for men and X71 for women. Sometimes symptom-based episode codes from the first visit might not be changed to the definitive gonorrhoea-ICPC after receiving laboratory outcomes. We expected that diagnoses at highest risk for missing gonorrhoea cases due to this missing recoding, were urethritis and epididymitis. Therefore, episodes coded as urethritis and epididymitis for men were collected and checked for a potential underlying gonorrhoea infection that was not recoded as gonorrhoea, respectively ICPC Y99 and Y74. The subcodes Y99.01 and Y99.02, respectively testicular torsion and spermatocele, were excluded.

**Fig. 1:**
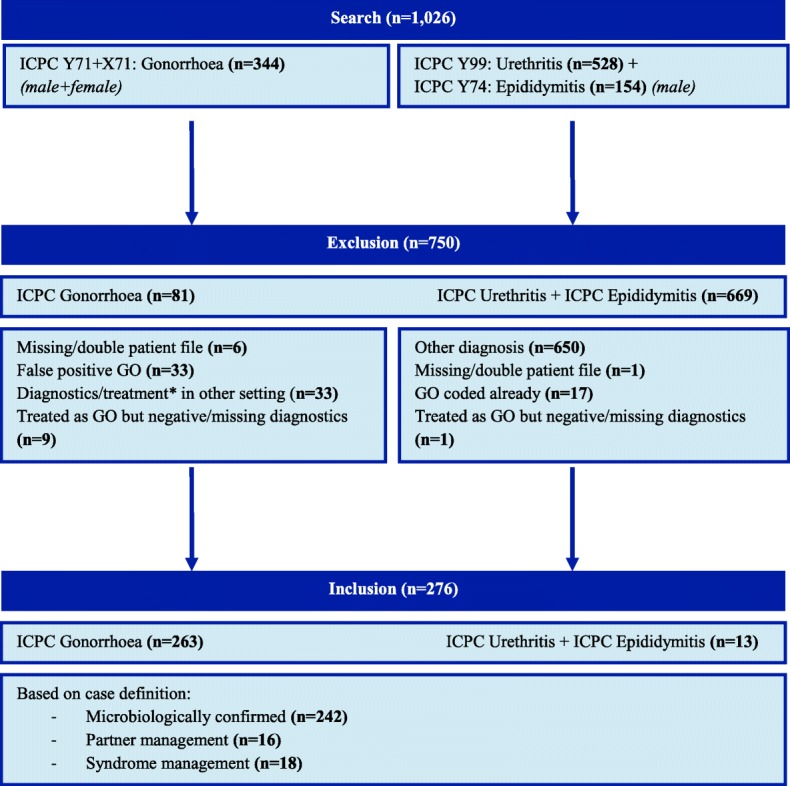
The inclusion of gonorrhoea cases based on ICPC codes

A period of 2 months before and 2 months after the respective diagnosis was determined as one episode. A case was defined as an episode confirmed to this period, including patients aged 15 years and older who received treatment in general practice. To assess the prescribing practices in the treatment of gonorrhoea, all cases were based on one of the three treatment indications applied in general practice according to the Dutch guideline: positive test outcome (nucleic acid amplification test, culture, gram stain), syndrome management (specific STI-symptoms) or partner management (a confirmed gonorrhoea infection in a sex partner) [6]. Cases that were diagnosed in other settings were excluded (Fig. 1 Inclusion of gonorrhoea cases based on ICPC codes). Details of all registered episodes were extracted together with the following case characteristics: date of birth, date of diagnosis, sex, anatomic location of infection, complication, coinfection (and if documented sexual orientation, ethnicity and HIV-status). An interval of 6 months after respective diagnosis was needed to check the HIV test results, because of the potential HIV window period [6].

### Survey design

The 35 GPs currently participating in the General Practice Department research database, concerning all six health centers, were approached by email with an online Dutch questionnaire to explore their experiences with the administration of intramuscular third-generation cephalosporins in the past years. Questions were designed by the authors and sent by head of the General Practice Department research database of the AUMC. Data were obtained on the opinion of three statements: 1) “I experience intramuscular administration of third-generation cephalosporins as a cumbersome procedure”, 2) “I prefer a single intramuscular dose over multiple oral doses (because of compliance)”, 3) “I prefer culture-based oral therapy over immediate blind intramuscular therapy”. The GP could indicate to what extent they were in agreement with each statement on a five point Likert scale. GPs were reminded once to complete the questionnaire.

### Data analysis

Descriptive analyses were performed using IBM SPSS Statistics version 23. The annual number and characteristics of diagnosed gonorrhoea infections were assessed. The incidence was calculated based on the yearly registered patients in the participating health centers in southeast-Amsterdam [14]. In addition, the current guideline implementation was demonstrated by reporting the annual percentage of administered first choice treatment according to the guideline. Since the updated guideline was published end of 2013, we defined intramuscular cefotaxime and ceftriaxone in the period 2010 to 2014 as first choice therapy in general practice. Since 2014 only ceftriaxone was defined as first choice treatment. The percentage and variety of alternative antibiotics were reported together with reasons for administration documented in the electronic patient files. This included the number of culture-based alternative antibiotic therapies.

### Ethics approval

The quantitative and qualitative data used for the study are anonymized. Therefore, formal assessment by the Medical Ethic Committee was not necessary according to Dutch.

## Results

### The diagnosis and incidence of gonorrhoea patients

**Fig. 2:**
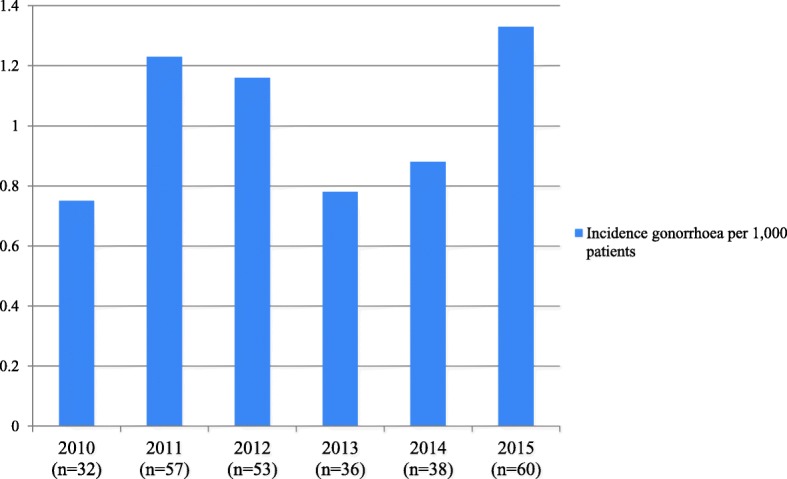
Incidence of all included gonorrhoea cases per year between 2010 and 2016

From 2010 until 2016, 1026 cases were checked for a potential gonorrhoea infection: 344 gonorrhoea and 682 urethritis or epididymitis coded cases. We excluded 750 cases based on incorrect coding, other diagnoses and missing details on diagnostics and treatment (assuming diagnostics and treatment have been performed in other settings). In total, 276 eligible cases could be included for final analyses. 263 cases coded as gonorrhoea and 13 cases coded as urethritis or epididymitis with documented treatment indications for a gonococcal infection were included (Fig. 1 Inclusion of gonorrhoea cases). The majority of the gonorrhoea cases were patients aged between 16 and 30 years and in 53% male (Additional file 1: Table S1). The annual incidence varied between 0.8 to 1.3/1000 patients over the whole period (Fig. 2 Incidence of all included gonorrhoea cases per year between 2010 and 2016, Additional file 2: Table S2).

The infections were microbiologically confirmed (positive NAAT, culture or gram stain) in 88% (242/276). In the remaining cases treatment was based on partner management (PM) or syndrome management (SM) without microbiological confirmation (*n* = 34). In 10 of these cases the test appeared negative after receiving treatment. The percentage of performed cultures was 36% (98/276). Forty-seven out of 54 (84%) negative culture outcomes were false negative based on a positive NAAT (Additional file 1: Table S1).

### Antimicrobial management

**Fig. 3:**
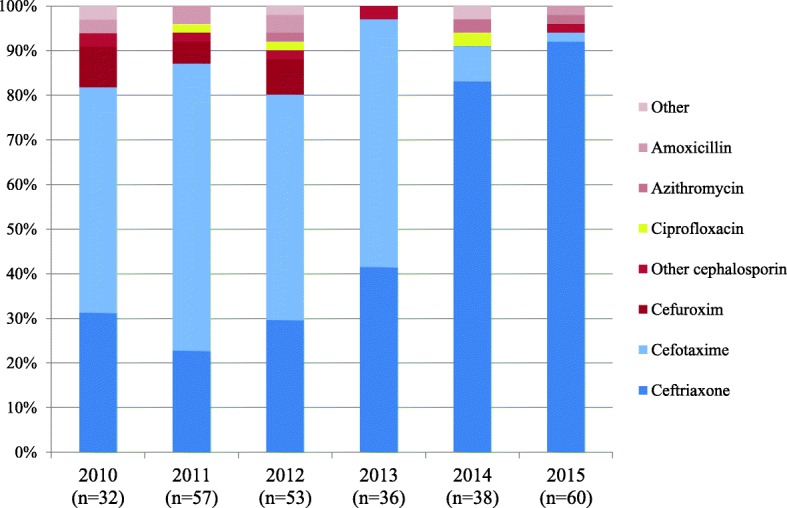
The antimicrobial management of gonorrhoea between 2010 and 2016

All the patients were treated with antibiotics. In the period 2010 until 2014 the number of gonorrhoea infections treated with the recommended antibiotics, either cefotaxime or ceftriaxone, increased from 81 to 97% (Fig. 3 The antimicrobial management of gonorrhoea between 2010 and 2016, Additional file 3: Table S3). In 2014 and 2015, 84 and 93% of the gonorrhoea infections respectively were treated with ceftriaxone monotherapy according to the updated national guideline. The percentage of ceftriaxone prescriptions increased significantly between 2010 and 2016 (*p* < 0.001). Between 2010 and 2016, three cases were treated with ciprofloxacin (based on culture outcomes). In 2015 none of the GPs prescribed ciprofloxacin for the treatment of gonorrhoea. In the whole period, only 34 patients (12%) received an alternative treatment (Additional file 3: Table S3). In 29% (10/34) of the alternative treated cases, the type of treatment was chosen because of one of the following argumentations: cultures with susceptibility patterns (*n* = 4), microbiologist consultation (*n* = 3), patient preference (*n* = 2) or unavailable recommended medication (*n* = 1). For the remaining alternative therapies argumentation could not be found or was not clear.

### Antibiotic treatment preferences

**Fig. 4:**
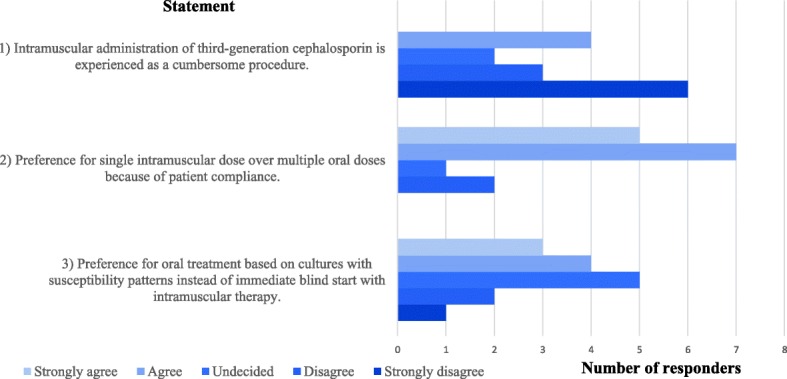
The GPs preferences in the treatment of gonorrhoea

34 GPs were eligible for the survey to assess experiences with the recommended therapy of intramuscular administered cephalosporins. A response rate of 43% (15/34) was noted. Sixty per cent (*n* = 9) of GPs disagreed or strongly disagreed that intramuscular administration of ceftriaxone is experienced as a cumbersome procedure (Fig. 4 The GPs preferences in the treatment of gonorrhoea). Eighty percent (*n* = 12) of the GPs agreed or strongly agreed with the preference of a single intramuscular dose over multiple oral doses because of patient compliance. The opinions regarding the last statement, concerning the preference for culture-based oral therapy instead of (immediate start of) blind intramuscular therapy, were diverse.

## Discussion

This study shows trends in antimicrobial management of gonorrhoea by GPs in the period from 2010 to 2016 in a high urban area, Amsterdam, the Netherlands. The number of gonorrhoea cases treated with recommended first choice therapy according to the guideline increased up to 93% in 2015. GPs did not experience barriers in the administration of the intramuscular third-generation cephalosporins.

The fact that 93% of the gonorrhoea cases were treated with the recommended ceftriaxone demonstrates a very high rate of compliance among the GPs in this study. These findings are in contrast with the rates of compliance in the treatment of gonorrhoea infections in general practice after publication of the first STI guideline, reported in the Dutch study mentioned earlier [11]. A possible explanation for the difference, could be that GPs participating in academic networks are more up to date with new developments and are earlier adopters of guidelines, compared to other GPs. In addition, the majority of the GPs at the General Practice network of the AUMC work in health centers with pharmacies which makes it easier to implement intramuscular therapy. Patients can collect their prescribed medicine in the same facility and have their injection delivered by the practice nurse.

Our data do differ from prescribing practices in primary care in other countries. It has recently been shown in Estonia that half of the gonorrhoea patients were not treated according to the recommended therapy by general practitioners (GPs) [15]. In the UK it has been demonstrated that GPs prescribed the recommended antibiotics for less than half of the gonorrhoea patients [16]. A survey in France revealed that only 20% of the GPs prescribed the recommended treatment for gonococcal urethritis, and ciprofloxacin was frequently used [17]. The GPs who had been in practice for a longer period and those who are older were less likely to adhere to the recommended treatment. In contrast to our findings, the authors hypothesized that the intramuscular administration of cephalosporins may hamper the recommended treatment.

### Limitations of the study

There are several limitations to the study. First, we studied a relative small sample size in a highly urban area with a high prevalence of gonorrhoea [13]. The prevalence of the disease, the background of the patients and the group of GPs are not representative of all areas of the nation. Furthermore, the limitations of routinely collected data should be noted. The ICPC-system is prone to unidentified coding errors, which may have affected the data. Finally, the questionnaire has been used to get an impression of the experiences of GPs with the recommended therapy. To draw more reliable and representative conclusions, a more validated survey including open-ended questions with higher response rate is needed.

## Conclusions

Given the threat of untreatable gonorrhoea, up-to-date guidelines with fast implementation and adherence in general practice are essential. Compared to the previous guideline implementation in the Netherlands, this study demonstrated a very successful shift in the treatment of gonorrhoea to a single intramuscular dose of ceftriaxone according to the national guideline and showed minimal barriers among GPs in a high prevalence area in Amsterdam between 2010 and 2016. Numbers are small, however, and more research into the prescribing patterns of GPs in a national representative sample is warranted.

## Additional files

Additional file 1: **Table S1.** Gonorrhoea patient characteristics. (DOCX 17 kb)

Additional file 2: **Table S2.** Trend analysis between time and number of gonorrhoea cases between 2010 and 2016. (DOCX 14 kb)

Additional file 3: **Table S3a.** Number of treated infections (including number of administered third-generation cephalosporins vs alternative drugs) between 2010 and 2016. **Table S3b.** Number of administrated drugs as treatment for gonorrhoea infections between 2010 and 2016. (DOCX 15 kb)

## Abbreviations

AUMC: Amsterdam University Medical Center,
EMR: electronic medical records
GP: general practitioner
GUM: genitourinary medicine
ICPC: International Classification of Primary Care-1
NAAT: nucleic acid amplification test
PM: partner management
SM: syndrome management
STI: sexually transmitted infections
WHO: World Health Organization
