# Supplementary material for: Trends in antimicrobial management of gonorrhoea by general practitioners in Amsterdam, the Netherlands, between 2010 and 2016: a cross-sectional study
Source: BMC Fam Pract. 2019 Jan 15;20:12. doi: 10.1186/s12875-018-0900-9 (PMC6332518; doi:10.1186/s12875-018-0900-9)
Supplement: Supplementary file 1 — Table S1. Gonorrhoea patient characteristics. (DOCX 17 kb) [file 12875_2018_900_MOESM1_ESM.docx]

| **Characteristics** | **2010-2015**  **N (%)** | **Missing**  **N (%)** |
| --- | --- | --- |
| **Gender:**   - **Male** - **Female** | 147 (53.3%) | 0 |
|  | 129 (46.7%) | 0 |
| **Age: (mean)**  **Diagnostic method:**   - **TOTAL documented** - **Microbiological confirmed (PCR/culture)** - **Syndrome management** - **Partner management** | 26.2  276 (100%) | 0 |
|  | 242 (87.7%) |  |
|  | 14 (5.0%) |  |
|  | 20 (7.2%) |  |
| **Anatomic location:**   - **TOTAL documented** - **Urogenital** - **Other/multiple locations*** | 254 (92.0%) | 22 (8.0%) |
|  | 243 (88.0%) |  |
|  | 11 (4.0%) |  |
| **Complication:**   - **TOTAL documented** - **Epididymitis** - **PID** - **Other**** | 12 (4.2%) | 264 (95.6%) |
|  | 4 (1.4%) |  |
|  | 5 (1.8%) |  |
|  | 3 (1.0%) |  |
|  |  |  |
| **Coinfection:**   - **TOTAL documented** - **Chlamydia trachomatis** - **Other/combination** - **Negative test** | 235 (85.1%) | 41 (14.9%) |
|  | 106 (38.4%) |  |
|  | 7 (2.5%) |  |
|  | 122 (44.2%) |  |
| **Sexual orientation:**   - **TOTAL documented** - **Heterosexual** - **MSM** - **Bisexual** | 70 (25.4%) | 206 (74.6%) |
|  | 61 (22.1%) |  |
|  | 6 (2.2%) |  |
|  | 3 (1.1%) |  |
| **Ethnicity:**   - **TOTAL documented** - **Dutch** - **Other** | 3 (1.1%) | 273 (98.9%) |
|  | 0 |  |
|  | 3 (1.1%) |  |
| **Culture**   - **TOTAL performed** - **Positive test** - **Negative test** - **False negative** | 98 (35.5%) | 178 (64.5%) |
|  | 44 (15.9%) |  |
|  | 54 (19.6%)  47 (84.3%) |  |
| **HIV-status:**   - **HIV+ before GO testing** | 15 (5.4%) | 261 (94.6%) |
| **Treatment:**   - **TOTAL documented** - **First choice** - **Alternative** | 276 (100%) | 0 |
|  | 242 (86.7%) |  |
|  | 34 (12.2%) |  |

** Other/combination location: oropharyngeal, anorectal.*

***Other complications: Bartholinitis, arthritis, prostatitis.*
