# Supplementary material for: Trends in antimicrobial management of gonorrhoea by general practitioners in Amsterdam, the Netherlands, between 2010 and 2016: a cross-sectional study
Source: BMC Fam Pract. 2019 Jan 15;20:12. doi: 10.1186/s12875-018-0900-9 (PMC6332518; doi:10.1186/s12875-018-0900-9)
Supplement: Supplementary file 2 — Table S2. Trend analysis between time and number of gonorrhoea cases between 2010 and 2016. (DOCX 14 kb) [file 12875_2018_900_MOESM2_ESM.docx]

| Variable | Gonorrhoea cases  (N) | | Logistic regression  95%CI | Logistic regression  P value | Chi square  P value |
| --- | --- | --- | --- | --- | --- |
| Time (in year):  2010  2011  2012  2013  2014  2015 | 34  57  53  36  39  60 | -0.989- -0.148  -0.446- 0.280  -0.512- 0.227  -0.953- -0.126  -0.823- -0.016  - | | **0.019**  0.008  0.653  0.449  0.011  0.042  - | 0.242 |
