# Supplementary material for: Trends in antimicrobial management of gonorrhoea by general practitioners in Amsterdam, the Netherlands, between 2010 and 2016: a cross-sectional study
Source: BMC Fam Pract. 2019 Jan 15;20:12. doi: 10.1186/s12875-018-0900-9 (PMC6332518; doi:10.1186/s12875-018-0900-9)
Supplement: Supplementary file 3 — Table S3a. Number of treated infections (including number of administered third-generation cephalosporins vs alternative drugs) between 2010 and 2016. Table S3b. Number of administrated drugs as treatment for gonorrhoea infections between 2010 and 2016. (DOCX 15 kb) [file 12875_2018_900_MOESM3_ESM.docx]

| Year | Treated gonorrhoea infections  (N) | Administered first choice treatment  N (%) | Administered alternative drugs  N (%) |
| --- | --- | --- | --- |
| 2010 | 32 | 26 (81%) | 6 (19%) |
| 2011 | 57 | 50 (88%) | 7 (12%) |
| 2012 | 53 | 43 (81%) | 10 (19%) |
| 2013 | 36 | 35 (97%) | 1 (3%) |
| 2014 | 38 | 32 (84%) | 6 (16%) |
| 2015 | 60 | 56 (93%) | 4 (7%) |

| Year | 2010 | 2011 | 2012 | 2013 | 2014 | 2015 |
| --- | --- | --- | --- | --- | --- | --- |
| Ceftriaxone | 10 (31.3%) | 13 (22.8%) | 16 (30.2%) | 15 (41.7%) | 32 (84.2%) | 56 (93.3%) |
| Cefotaxime | 16 (50.0%) | 37 (64.9%) | 27 (50.9%) | 20 (55.6%) | 3 (7.9%) | 1 (1.7%) |
| Cefuroxime | 3 (9.4%) | 3 (5.3%) | 4 (7.5%) |  |  |  |
| Other cephalosporin | 1 (3.1%) | 1 (1.8%) | 1 (1.9%) | 1 (2.8%) |  | 1 (1.7%) |
| Ciprofloxacin |  | 1 (1.8%) | 1 (1.9%) |  | 1 (2.6%) |  |
| Azithromycin 1g  Azithromycin 2g |  |  | 1 (1.9%) |  | 1 (2.6%) | 1 (1.7%) |
| Amoxicillin | 1 (3.1%) | 2 (3.5%) | 2 (3.8%) |  |  | 1 (1.7%) |
| Other/combination³ | 1 (3.1%)¹ |  | 1 (1.9%) |  | 1 (2.6%) |  |
